# Frostbite: a practical approach to hospital management

**DOI:** 10.1186/2046-7648-3-7

**Published:** 2014-04-22

**Authors:** Charles Handford, Pauline Buxton, Katie Russell, Caitlin EA Imray, Scott E McIntosh, Luanne Freer, Amalia Cochran, Christopher HE Imray

**Affiliations:** 1 Queen Elizabeth Hospital, Birmingham B15 2TH, UK; 2 University Hospital Coventry & Warwickshire, Coventry CV2 2DX, UK; 3 University of Utah, Salt Lake City, UT 84112, USA; 4 Sheffield University Medical School, Sheffield S10 2TN, UK; 5 Division of Emergency Medicine, University of Utah Health Care, Salt Lake City, UT 84112, USA; 6 FAWM, Yellowstone National Park, WY 82190, USA; 7 Everest ER, Himalayan Rescue Association, P.O. Box 4944, Kathmandu 44601, Nepal; 8 University of Utah School of Medicine, Salt Lake City, UT 84132, USA; 9 Warwick Medical School, The University of Warwick, Coventry CV4 7AL, UK; 10 University Hospital Coventry and Warwickshire NHS Trust, Coventry CV2 2DX, UK

**Keywords:** Frostbite, Hypothermia, Rewarming, Thrombolysis, Heparin, TPA, Iloprost

## Abstract

Frostbite presentation to hospital is relatively infrequent, and the optimal management of the more severely injured patient requires a multidisciplinary integration of specialist care. Clinicians with an interest in wilderness medicine/freezing cold injury have the awareness of specific potential interventions but may lack the skill or experience to implement the knowledge. The on-call specialist clinician (vascular, general surgery, orthopaedic, plastic surgeon or interventional radiologist), who is likely to receive these patients, may have the skill and knowledge to administer potentially limb-saving intervention but may be unaware of the available treatment options for frostbite. Over the last 10 years, frostbite management has improved with clear guidelines and management protocols available for both the medically trained and winter sports enthusiasts. Many specialist surgeons are unaware that patients with severe frostbite injuries presenting within 24 h of the injury may be good candidates for treatment with either TPA or iloprost. In this review, we aim to give a brief overview of field frostbite care and a practical guide to the hospital management of frostbite with a stepwise approach to thrombolysis and prostacyclin administration for clinicians.

## Review

### Introduction

Frostbite is a freezing, cold thermal injury, which occurs when tissues are exposed to temperatures below their freezing point (typically −0.55°C, but can occur as high as 2°C) for a sustained period of time [1]. It is a condition that has far-reaching consequences in terms of functional morbidity to a population that are often young, fit and healthy prior to the thermal injury. Many frostbite patients in urban areas are homeless and/or suffer from mental health issues. Frostbite is well documented in the military and in countries with extreme temperatures for centuries. The earliest documented evidence of frostbite may be a 5,000-year-old pre-Columbian mummy discovered in the Andes [2]. The first report of mass cold injuries was by Baron Larrey, Surgeon-in-Chief to Napoleon's Army throughout the invasion of Russia during the winter of 1812–1813 [3]. Larrey introduced the concept that the physiologic response to cold injuries was similar to that of burn injuries and recognized that warming frozen tissue was advantageous for recovery.

Today, the presentation of frostbite is increasing within the civilian population, in particular those who partake in winter sports such as skiing, hiking, mountain and ice climbing [4]. The outdoors is more accessible, and individuals with limited experience/inadequate preparation and protection find themselves at risk of cold thermal injury [5]. Vagrancy, homelessness, industrial injury and malfunctioning or misuse of equipment using NO or CO_2_ have also been described [6,7].

Severity of injury depends on factors such as absolute temperature, wind chill, duration of exposure, wet/dry cold, immersion, clothing quality and patient comorbidities such as smoking, peripheral vascular disease, neuropathies, Raynaud's disease, mental health issues, substance abuse and dementia [1,4,8,9]. Alcohol consumption is potentially particularly devastating as it causes heat loss through peripheral vasodilatation and also impairs judgement. This may affect the individual's ability to seek adequate shelter, compounding the injury. Amputation of injured parts has been shown to correlate more closely with the duration of cold exposure rather than temperature [9]. Anatomically, the hands and feet account for 90% of reported injuries [10,11]. Frostbite can also affect the face (nose, chin, earlobes, cheeks and lips), buttocks/perineum (from sitting on metal seats) and penis (joggers and Nordic skiers). Patients at the extremes of age (elderly and infants/young children) are at greater risk because of immobility and higher surface area-to-mass ratio (children); however, studies show that frostbite is uncommon in these age groups and instead is seen more commonly in adults between the ages of 30 and 49 years, most likely due to increased exposure to cold or risk-taking behaviour [10,11].

Frostbite can result in a wide spectrum of injury, ranging from complete resolution without significant sequelae to major limb amputation and its functional consequences. Once in the hospital setting, the best outcomes will be achieved for the patient when a multidisciplinary approach is utilized [11]. In this practical guide, we review key current frostbite literature, classification strategies and recommendations for management of frostbite in the hospital setting.

#### Literature search

A systematic literature search of the related articles published between January 1969 and July 2013 was performed using PubMed (restricted to the English language) with keywords ‘frostbite’, ‘frostbite management’ and ‘freezing cold injury’. The search included both human and animal studies, original research, case series/reports, review articles and guidelines. Priority was given to human studies and more recent publications since 2005. The studies were identified by title and abstract and screened by the authors; relevant cross-references were added.

#### Recommendation grade

Using the criteria defined by the American College of Chest Physicians (ACCP), each form of intervention was attributed a recommendation grade where appropriate. For further details on the criteria, please refer to Table 1[12].

**Table 1:** **ACCP classification criteria for grading evidence in clinical guideline**[13]

**Grade**	**Description**	**Benefits vs. risks and burdens**	**Methodological quality of supporting evidence**
1A	Strong recommendation, high-quality evidence	Benefits clearly outweigh risks and burdens or vice versa	RCTs without important limitations or overwhelming evidence from observational studies
1B	Strong recommendation, moderate-quality evidence	Benefits clearly outweigh risks and burdens or vice versa	RCTs with important limitations or exceptionally strong evidence from observational studies
1C	Strong recommendation, low-quality or very low-quality evidence	Benefits clearly outweigh risks and burdens or vice versa	Observational studies or case series
2A	Weak recommendation, high-quality evidence	Benefits closely balanced with risks and burdens	RCTs without important limitations or overwhelming evidence from observational studies
2B	Weak recommendation, moderate-quality evidence	Benefits closely balanced with risks and burdens	RCTs with important limitations or exceptionally strong evidence from observational studies
2C	Weak recommendation, low-quality or very low-quality evidence	Uncertainty in the estimates of benefits, risks and burden; benefits, risk and burden may be closely balanced	Observational studies or case series

RCT, randomized controlled trial.

#### Pre-hospital care and prevention

Prevention is always preferable, and education of those working or recreating in cold environments should focus on modification of risk factors, selection and use of proper clothing, optimal nutrition and hydration [4]. Those working with equipment that uses coolant such as liquid nitrogen or carbon dioxide should also have adequate education in safe handling of such products.

Whilst pre-hospital care is not the focus of this article, key field management of frostbite concepts are summarized in the following texts [13,14]. In general, the patient should be moved out of the wind, provided with shelter and be given warm fluids (*recommendation grade 1C*).

Remove boots (but consider problems of replacement if swelling occurs), and replace wet gloves and socks with dry ones. Warm the cold extremity by placing it in a companion's armpit or groin for 10 min and then replace the boots/gloves. Rubbing the affected part is not recommended because of the potential for worsening direct tissue injury (*recommendation grade 1C*).

If sensation returns, the patient may mitigate risks (e.g. add a layer and change to warmer or dryer socks or boots) and continue to walk. If there is no return of sensation, the injured should go to the nearest warm shelter (hut or base camp) and seek medical treatment. If at high altitude (>4,000 m), supplementary oxygen should be considered [11] (*recommendation grade 2C*).

Aspirin 75 mg can be given for its rheologic effect. Ibuprofen 12 mg/kg/day divided into two daily doses (maximum of 2,400 mg/day) should be given for its prostaglandin effect (*recommendation grade 2C*).

Field rewarming should only be attempted if there is no further risk of refreezing [14,15]. Tissue that thaws then refreezes results in more extensive injury (*recommendation grade 1B*).

The decision to thaw the frostbitten tissue in the field commits to a course of action that may involve pain control, maintaining warm water baths at a constant temperature, protecting tissue from further injury during rewarming and eventual transport. In extreme circumstances, it may be better to let a casualty walk on a frozen limb to safety rather than risk refreezing [16] (*recommendation grade 1C*).

### Hospital management

#### Immediate and general care for those admitted with frostbite

On arrival to a hospital setting, it is vitally important to fully reassess the patient. Underlying unstable comorbidities, trauma or hypothermia must be assessed and managed before frostbitten extremities are treated. Moderate or severe hypothermia should be corrected to bring core temperature above 35°C before initiating frostbite warming [14,17] (*recommendation grade 1C*).

A detailed history should include time the injury occurred, either early (<24 h) or late (>24 h) as this will dictate some treatment options. History of the conditions surrounding the injury (i.e. temperature, wind chill, wet/dry exposure, duration and use or not use of thermal protection) can also be helpful. Any pre-hospital treatment and time of rewarming, if applicable, should be noted [14].

**Figure 1:**
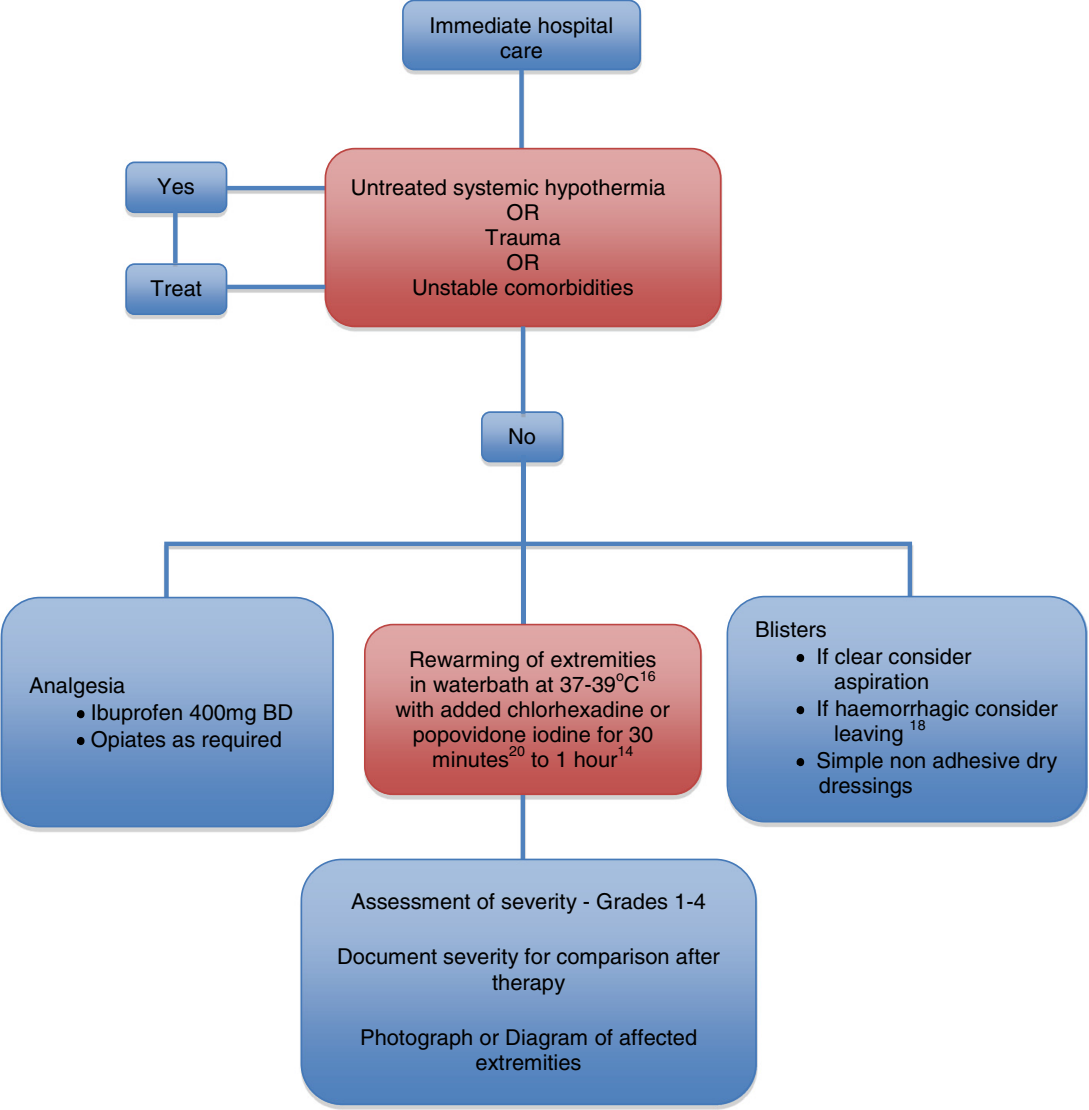
Immediate hospital management of frostbite injury.

Remove jewellery from affected digits early as significant swelling can be expected post thaw, and vascular compromise may occur with tight rings, etc. [14]. Examination of the frostbitten tissue after rewarming can predict depth of injury more accurately than examination before thawing. There may be different depths of injury even on digits of the same extremity, so careful examination and documentation in either diagram form or with photographs are useful. Clinical photography obviates the need for repeated removal of dressings for each consultant examination, reducing pain and risk of infection. Loss of sensation after rewarming is a poor prognostic indicator, and the converse is also true. Figure 1 suggests how one should proceed with initial in hospital management.

#### Classification

There exist a number of frostbite classifications to assess the severity and predict likely outcome. Cauchy et al. have suggested a useful classification consisting of four grades and three key descriptors (Table 2) [18]. At 24 h post insult after rewarming, a grade can be attributed according to the level of any visible lesion. Then, at day two, a technetium^99^ triple-phase bone scan should be performed on the more severe injuries (see Imaging section) and a further assessment of any blisters undertaken. Injuries receiving grade 1 classification require no hospitalisation and full recovery is likely. Grades 2 through 4 injuries require hospitalisation and full investigation as they are associated with an increased risk of amputation and long-term sequelae [18].

**Table 2:** **Classification scheme for the severity of frostbite injury**[19]

**Frostbite injuries of the extremity**	**Grade 1**	**Grade 2**	**Grade 3**	**Grade 4**
Extent of initial lesion at day 0 after rewarming	Absence of initial lesion	Initial lesion on distal phalanx	Initial lesion on intermediary (and) proximal phalanx	Initial lesion on carpal/tarsal
Bone scanning at day 2	Useless	Hypofixation of radiotracer uptake area	Absence of radiotracer uptake on the digit	Absence of radiotracer uptake area on the carpal/tarsal region
Blisters at day 2	Absence of blisters	Clear blisters	Haemorrhagic blisters on the digit	Haemorrhagic blisters over carpal/tarsal region
Prognosis at day 2	No amputation	Tissue amputation	Bone amputation of digit	Bone amputation of the limb
	No sequelae	Fingernail sequelae	Functional sequelae	+/− systemic involvement
				+/− sepsis functional sequelae

#### Fluids

Rehydration can be oral or intravenous, and depending upon severity and ability of the patient to tolerate oral fluids. High altitude increases the risk of dehydration. If the patient is also hypothermic, dehydration may be compounded by cold diuresis due to suppression of antidiuretic hormone, requiring correction with warmed intravenous fluids (*recommendation grade 1C*).

#### Rewarming

Rewarming is beneficial if there remains a partially or fully frozen part and is ideally accomplished using a whirlpool bath set at 38°C with added antiseptic solution (povidone iodine or chlorhexidine). The Wilderness Medical Society and State of Alaska Cold Injury Guidelines recommend a temperature of 37°C–39°C, which decreases the pain experienced by the patient whilst only slightly slowing rewarming time [14,15]. The time period for optimal rewarming varies from 15–30 min up to 1 h [16]. Rewarming should continue until a red/purple colour appears and the extremity tissue becomes pliable [14]. Active motion during the rewarming process is beneficial, but care should be taken to prevent the extremity from touching the sides of the whirlpool. It is important to provide good analgesic cover and is likely to include narcotic medication (*recommendation grade 1B*).

#### Blisters and dressings

It is important to note the type of any blisters that form; they can be clear/cloudy or haemorrhagic in nature. There is current debate as to whether blisters should be de-roofed as this may desiccate the underlying tissue, but there is little comparative data to settle this argument. The recommended practice is that of selective drainage of clear/cloudy blisters by needle aspiration (especially if bullae restrict movement) and to leave haemorrhagic blisters alone [14]. However, we would suggest that all blisters are debrided in the hospital (not in the field) because we believe it assists with wound care. Severe injuries require detailed assessment, and it may be that this appraisal and debriding of blisters may be best performed under a general anaesthetic (*recommendation grade 2C*).

Topical aloe vera cream or gel (a potent anti-prostaglandin agent) should then be applied to thawed tissue before dressings are applied [14] (*recommendation grade 2C*). Splinting, elevating and wrapping the affected part in a loose, protective dressing with padding between affected patient's digits are ideal (*recommendation grade 1C*).

#### Antibiotics

The role of prophylactic antibiotics is not proven but should be considered in more severe injuries (grades 3 and 4) and, in particular, when associated with significant oedema or malnutrition (homeless, chronic alcohol abuse or return from extreme altitude). Systemic antibiotics are required in the presence of proven infection, trauma or cellulitis (*recommendation grade 1C*).

#### Tetanus toxoid

The need for tetanus toxoid administration should be determined by following standard guidelines, as frostbite injuries are not inherently tetanus-prone wounds (*recommendation grade 1C*).

#### Analgesia and NSAIDs

Rewarming the extremities can become extremely painful, so use of non-steroidal anti-inflammatory drugs or opiates should be administered. Oral ibuprofen 12 mg/kg divided over two daily doses provides systemic anti-prostaglandin activity that limits the cascade of inflammatory damage. This dose can be increased to a maximum of 2,400 mg/day if the patient is experiencing pain and can be continued until wounds are healed or amputation occurs. A dose of 400 mg BID is a practical regime on which to start most patients, and this can then be increased to 600 mg QDS as pain dictates. If aspirin has not been given in the field (providing no contraindications), 300 mg once a day can be given [4] (*recommendation grade 2C*).

### Management specific to frostbite

For more superficial injuries, often, no more intervention or investigation is required after basic treatment has been initiated (Cauchy and Chetaille grade 1); however, in more severe cases, further intervention is required. Advanced imaging may be used to determine depth of tissue injury and guide therapy. It will also give an accurate prognosis at an early stage as to the subsequent likely clinical course. This is important for the patient, clinicians and occasionally for medico-legal reasons.

#### Imaging

For deep injuries, no surgical debridement should be planned until imaging is performed. Many modalities have been used, but angiography and technecium^99^ (^99^Tc) triple-phase bone scanning give the best prognostic information and will direct therapy [1] (*recommendation grade 1C*).

A retrospective review of 92 patients with severe frostbite by Cauchy et al. [19] showed that ^99^Tc scans obtained 2 days after the injury accurately predicted the level of amputation in 84% of cases. ^99^Tc scanning has been performed on the day of presentation [20]. Case reports suggest magnetic resonance angiography (MRA) superiority to ^99^Tc as it allows direct visualization of occluded vessels and surrounding tissue and may show a clearer demarcation of ischaemic tissues, but this has yet to be confirmed by larger studies [21]. However, MRA is easier to access in many units, and there appears to be a growing trend of using MRA as an alternative imaging technique.

#### Angiography and thrombolysis

A screening and treatment tool has been proposed for the use of thrombolytics in frostbite (see Table 3) [17].

**Table 3:** **A proposed screening and treatment tool for the use of thrombolysis in cases of frostbite**[17]

	**Questions/indications to be considered**
Treatment screen (four ‘yes’ answers required to proceed to angiography)	Are the patient's gas exchange and haemodynamics stable?
	Is flow absent after rewarming (no capillary refill or Doppler signals)?
	Was the cold exposure time less than 24 h?
	Is the warm ischaemia time less than 24 h?
Treatment protocol	Perform angiography with intra-arterial vasodilators
	If there is still no flow after angiography with vasodilators, infuse tissue plasminogen activator (rTPA) with systemic heparinization with priority to the hands; other sites receive a systemic dose
	Repeat angiography after 24 h
Indications for stopping the infusion of the rTPA	When restored flow has been confirmed by angiography or clinical examination
	If major bleeding complication occurs
	After 72-h treatment
Post lysis anticoagulation	One month of subcutaneous low-molecular weight heparin at prophylactic dose

**Figure 2:**
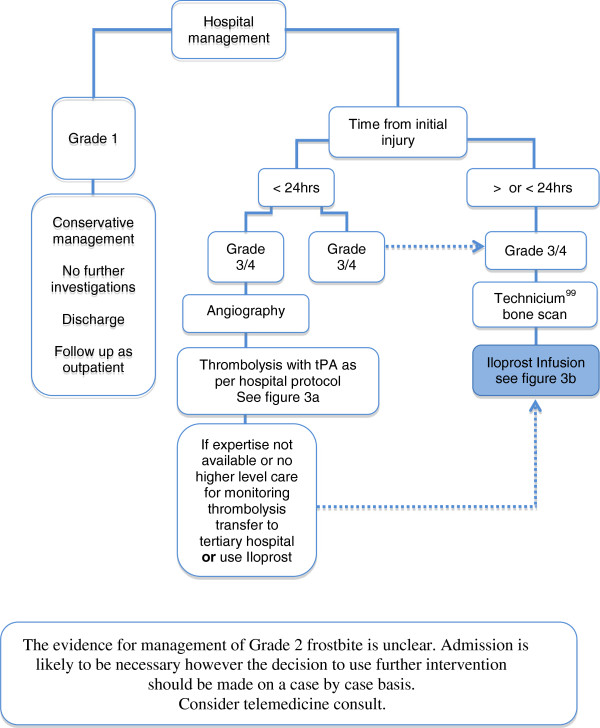
Algorithm for the use of rTPA and iloprost in the management of frostbite injuries.

An initial selective diagnostic digital subtraction angiography should be performed in patients being considered for thrombolysis. Intravenous vasodilators (nitroglycerin or papaverine) are useful (in conjunction with TPA) at this stage in the treatment of the vasospasm that often accompanies a frostbite injury [17,22,23]. It is possible that non-invasive MRA may offer a suitable alternative imaging modality (Figure 2).

In animal models, intravenous streptokinase limited the extent of tissue damage in a hind limb of a rabbit [24]. Streptokinase treatment and rapid rewarming resulted in reduced tissue damage and was most beneficial when given within 12 h of freezing and was still effective even when treatment was delayed up to 48 h [24].

Twomey et al. published results of an open-label study to evaluate the safety and efficacy of tissue plasminogen activator (rTPA) in the treatment of severe frostbite found that rTPA and heparin after rapid rewarming is safe and reduced predicted digit amputations. Similar efficacy was reported in both the intravenous and intra-arterial delivery arms [25]. Those patients with more than 24 h of cold exposure, warm ischaemia times greater than 6 h or evidence of multiple freeze-thaw cycles were least likely to benefit [25].

Bruen et al. demonstrated a reduction in digital amputation rates from 41% in those patients that did not receive rTPA to 10% in those receiving rTPA within 24 h of injury (*p* < 0.05) [26]. It was also noted that efficacy after 24 h decreased. Thrombolysis within 24 h (early group) appears to show the best outcomes in digit salvage [17]; however, thrombolysis after 24 h should be considered on an individual risk-benefit basis.

Delivery of rTPA can be either intravenous (IV) or via catheter-directed intra-arterial (IA) administration [17,25,26]. The latter is our preferred route, if rTPA is to be used rather than iloprost. Normal contraindications to TPA apply including existing trauma, recent surgery, neurological impairment or bleeding diathesis. It is not appropriate for superficial frostbite (grade 1), only deep tissue injuries that affect more proximal phalanges and the forefoot or foot should be considered, as treatment is not without risks of haemorrhage [17].

rTPA delivery should be provided at a centre accustomed to performing thrombolysis and that can provide adequate monitoring (usually in a critical care/high-dependency setting). If the patient presents less than 24 h after injury to a hospital without these facilities, consider urgent transfer in order not to delay commencement of therapy. rTPA is used in combination with heparin, which reduces the recurrence of microvascular thrombosis.

**Figure 3:**
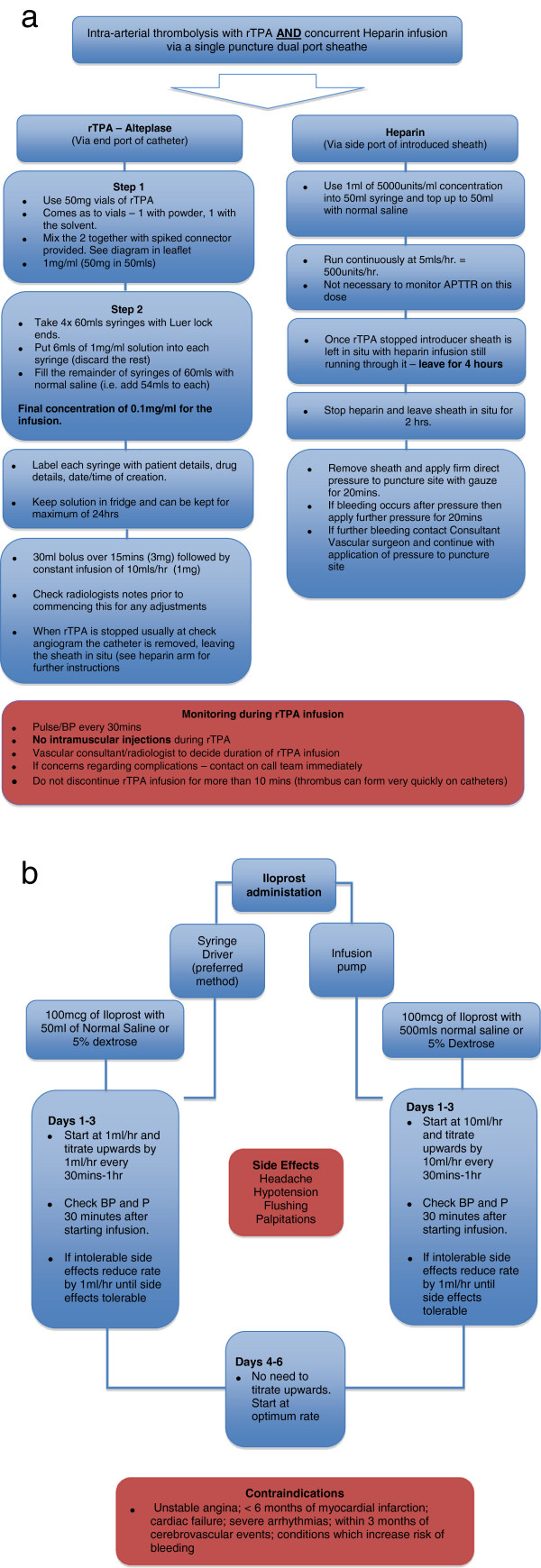
**Intra-arterial administration of rTPA and heparin and administration of intravenous iloprost. (a)** Algorithm for the intra-arterial administration of tTPA and heparin for in-hospital thrombolysis of severe frostbite injury. **(b)** Algorithm for the administration of intravenous iloprost for in-hospital thrombolysis of severe frostbite injury.

Repeat angiograms should be performed every 12–24 h to evaluate response to therapy. rTPA treatment should be discontinued when perfusion is restored to distal vessels or at 48 h if no improvement is observed [26]. Figure 3a gives a stepwise approach to intra-arterial thrombolysis (*recommendation 1B-C*).

#### Iloprost

Iloprost is a prostacyclin analogue with vasodilatory properties that mimic the effects of a sympathectomy [27]. It may also affect platelet aggregation and therefore decrease microvascular occlusion. Unfortunately, intravenous iloprost is not currently available in the USA.

In 1994, Groechenig published his experience in treating four cases of severe frostbite with iloprost [28]. The results were promising, with no patients requiring amputation; however, since these initial findings were published, the focus has shifted towards rTPA, with no further data published on iloprost use until a recent paper by Cauchy et al [29]. In a randomized controlled trial designed to compare the efficacy of iloprost and rTPA, 47 patients were included with a total of 407 digits at risk. All patients underwent identical initial treatment and rewarming and then were randomized into three arms: buflomedil, iloprost or iloprost and IV rTPA. The risk of amputation on the buflomedil arm was the greatest with 39.9% of at-risk digits requiring amputation. In the iloprost and iloprost/rTPA arms, the amputation rates were 0% and 3.1%, respectively [29].

The administration of iloprost is via an IV infusion. The dose used is 0.5 up to a maximum of 2 ng/kg/min [29], incrementally increased every 30 min by 0.5 ng/kg/min until the patient develops unacceptable or intolerable side effects (headache and hypotension). The rate is then reduced by 0.5 ng/kg/min. The infusion is continued for 6 h/day for 5–8 days at the previously determined maximal rate a patient can tolerate.

The advantages of iloprost compared to rTPA are that it does not require radiological intervention during administration and can be managed on a general or vascular ward. Iloprost can be used when there is a history of trauma or when the exposure occurred over 24 h ago, unlike rTPA where trauma is a contraindication and efficacy is reduced beyond 24 h. Figure 3b gives a stepwise approach to iloprost administration (*recommendation grade 1B*).

#### Surgery

Immediate amputation should be avoided; there is rarely any need for early intervention unless there is wet gangrene, liquefaction, overwhelming infection or spreading sepsis [30,31]. Planning is vital with a goal of obtaining the best functional outcome. Premature amputation increases morbidity and is likely to lead to poor subsequent function (*recommendation grade 1C*).

Fasciotomies are occasionally required post thaw if reperfusion is compromised by compartment syndrome [31] (*recommendation grade 1C*). The majority of amputations can be performed 6–12 weeks post injury once demarcation of ischaemic tissue has been well defined [4]. Negative pressure devices can aid in speeding up healing of amputation sites when left to heal by secondary intention [32].

#### Tissue protection

During the demarcation period, it is important to provide adequate protection especially footwear. Therefore, liaison with orthotic/podiatry department to provide bespoke footwear that protects and also attempts to maintain limb function is vital.

Following amputation, function is variable and injury specific. The biomechanics of the foot/hand can be radically altered and frostbite neuropathy can compound the problem; so, again, custom-made footwear may be required to optimize the long-term functional result and minimize secondary injuries [33].

#### Adjunctive therapies

The below therapies have insufficient trials performed to present any cognizant argument for their use but have been described in case reports or animal studies.

##### Hyperbaric oxygen therapy

By increasing oxygen tension in the blood, more oxygen is delivered to the tissues; however, this requires patent microvasculature. Hyperbaric oxygen therapy (HBOT) increases the deformability of erythrocytes, diminishes oedema formation in burns and post ischaemic tissues and has some bacteriostatic properties [34].

HBOT in frostbite has had mixed results with no level 1 evidence available. Animal studies have demonstrated no benefit [35], yet two recent human case series have yielded excellent results [34,36]. Significant thrombosis of the microvasculature may be the cause of its variable effect. Thus, currently, there is insufficient data to recommend its routine use (*no recommendation grade due to insufficient evidence*).

##### Sympathectomy

Surgical or chemical sympathectomy has yielded mixed results in improving blood flow. Early sympathectomy performed within the first few hours of injury is said to increase oedema formation and, consequently, tissue loss; however, if performed 24–48 h after thawing, resolution of oedema and decreased tissue loss are observed [37].

Sympathectomy may have a role in managing long-term sequelae of frostbite such as pain (often due to vasospasm), paraesthesias and hyperhidrosis [37]. However, since sympathectomy is irreversible, great caution should be exercised when considering its use, given the availability of alternative IV vasodilators [11] (*no recommendation grade due to insufficient evidence*).

#### Long-term management

The long-term sequelae of frostbite are less well studied. However, it is known that the tissue, which has recovered from frostbite, may be more susceptible to subsequent freezing injury. Consequently, patients should be educated about this risk especially if they plan to return to cold environments.

A long-term follow-up study of 30 patients with significant frostbite injuries showed that 53% exhibited cold hypersensitivity, 40% numbness of the digits and 33% had reduced sensitivity to touch [37]. The study postulates that these side effects may be secondary to a thermo-physiological response with an increased tendency to vasospasm. With this cold sensitization, the individual may be unable to tolerate cold temperature upon the previously frostbitten area, even when other body areas are comfortable at that temperature [38].

Chronic regional pain is perhaps the most common complaint post frostbite. The pain is often unresponsive to conventional analgesia and may be lifelong. Medications such as amitriptyline or gabapentin may have some benefit, but referral to a chronic pain specialist should be made in these cases.

Localized osteoporosis and sub-chondral bone loss can be seen post injury and reflect the severity of vascular damage. Changes can be seen within a month of injury but often progress over months such that by 16 months, radiographs can reveal multiple lucencies in the sub-chondral bone [39]. In children, the damage may be more significant with undergrowth of affected bone and development of early arthritis [39,40].

Skin areas that have been affected by frostbite are susceptible to chronic ulceration due to poor tissue quality after healing and can undergo a malignant transformation akin to the formation of Marjolin's ulcers observed in old burn scars [41].

#### Accessing expert advice (telemedicine)

Patients and clinicians with limited experience of frostbite can now use the internet and satellite phones to access expert advice in remote or difficult situations. A virtual opinion or more specialized advice can be sought from almost anywhere in the world using a combination of digital images and telephone advice [4,11,42,43].

## Conclusions

Deep frostbite is a serious condition that is associated with significant morbidity, and it is becoming more frequent in young active individuals who put themselves at risk. Timely pre-hospital and definitive hospital management are important to minimize final tissue loss and maximize functionality of the affected limb.

Surgeons should not to rush to early amputation; if managed correctly in the first few days, significant tissue can be salvaged, which is very important to the final functional outcome. We have outlined a series of management frameworks, which we hope will enable surgeons who rarely see this condition to have a greater understanding of frostbite and its management.

Either intravenous iloprost or thrombolysis with rTPA should be considered in all patients who present within 24 h of sustaining an appropriately severe injury and if the facility is capable of appropriate administration and monitoring. Both treatments should be started as soon as it is practical to gain maximal benefit. There is some evidence iloprost can be used beyond the 24 hour window and it is the treatment of choice where there are contraindications to thrombolysis. Bone scanning is helpful to ascertain deep tissue injury and response to therapy.

If iloprost is an available option (and it is not currently available in USA), then iloprost is the preferred option based upon its simplicity of administration, safety and efficacy. Prevention with education, behaviour modification and appropriate use of suitable equipment is important to reduce frostbite incidence.

## Abbreviations

C: centigrade; HBOT: hyperbaric oxygen therapy; IA: intra-arterial; IV: intravenous; kg: kilogramme; m: metres; mg: milligramme; MRA: magnetic resonance angiography; rTPA: recombinant tissue plasminogen activator; 99Tc: technetium-99.

## Competing interests

The authors declare that they have no competing interests.

## Authors’ contributions

CH and CHEI have done the conception and design of the study and the writing and final approval of the manuscript. PB wrote the manuscript and approved the final manuscript. KR, SM, LF and AC were involved in manuscript writing and in its critical revision and final approval. CEAI wrote and approved the final manuscript. All authors read and approved the final manuscript.
